# Investigating Noise Exposure to Newborn Infants From Respiratory Support: Methodological Considerations

**DOI:** 10.7759/cureus.19353

**Published:** 2021-11-08

**Authors:** Devika Singh, Gerhard Fusch

**Affiliations:** 1 Department of Pediatrics, McMaster University, Hamilton, CAN; 2 Department of Health Research Methods, Evidence and Impact, McMaster University, Hamilton, CAN

**Keywords:** neonatal intensive care unit (nicu), patient safety, ventilation, newborn, noise

## Abstract

Background and objective

Excessive noise in the neonatal intensive care unit (NICU) may lead to serious long-term effects on hearing and sensory development in newborns. As such, the maximum allowed noise level is 45 A-weighted decibels (dBA). Studies regarding noise exposure to ventilated preterm infants show inconsistent results; however, these studies also vary considerably in their methodology in terms of noise ascertainment. We hypothesized that the study methodology can significantly influence data quality when measuring noise levels. In this study, we aimed to investigate whether the variations in ventilator noise levels in NICUs could be a result of methodological differences in study designs.

Methods

A ventilator circuit was set up using nasal continuous positive airway pressure (nCPAP) and high-frequency (HF) modes with nasal prongs. Noise levels were measured using a commercially calibrated noise meter. Three different scenarios were tested: (1) measurements were taken at different angles (0° to 180°), with 180° facing the end of the nasal prongs, without a mannequin, with the membrane/orifice of the noise meter placed 2 mm laterally from the prongs; (2) noise levels were measured at 180° at distances of 0-20 mm from the nasal prongs; (3) measurements were taken in the oral cavity of a life-size intubation mannequin of a newborn baby.

Results

Overall, the noise levels produced at different settings varied significantly, ranging from 45.7 dB to 82.2 dB. The average environmental background noise was 44.4 dB. Noise levels typically increased as the angle increased, with the highest noise level recorded at 180° for both HF and nCPAP modes, at 58.4 dB and 58.2 dB, respectively. Noise levels recorded at HF were slightly higher than nCPAP values. Furthermore, with regard to distance, the highest mean value, 82.2 dB, was recorded with the noise meter approximately 3 mm from the nasal prongs, and the lowest mean value, 47.6 dB, was recorded at ~20 mm. During trials with the mannequin, the lowest value, 50.1 dB, was recorded at the entrance of the mouth with slightly higher values being recorded within the oral cavity.

Conclusion

The results indicate that small changes in experimental settings, such as positioning and distance from the nasal prongs, can greatly influence noise levels, particularly above the recommended levels for neonates. These differences may be attributed to wind-generated noise. In summary, some study results are potentially influenced more by the study design than the device type or ventilator setting. We recommend further research and detailed reporting in the NICU to gain deeper insights into the topic.

## Introduction

Infants in neonatal intensive care units (NICUs) are regularly exposed to noise from people conversing, and devices, such as alarms and ventilators. Often, noise levels exceed the recommended maximum average of 45 dB [1,2]. Previous studies have shown that excessive noise can lead to adverse effects on preterm infants’ respiratory and cardiovascular systems, as well as effects on hearing and sensory development [3-5]. The level needed to cause injury occurs when an individual is exposed to 90 dB for at least four hours [2]. While many studies have demonstrated that ventilator noise exceeds recommended levels, there are still discrepancies in reported results, which may be due to differences in experimental settings.

Previous literature has demonstrated that measured noise levels ranged from 39.5 dB to 89.2 dB [6-14]. All but one study has argued that noise levels above 45 A-weighted decibels (dBA) were above the maximum recommended noise level [2]. Studies that used different equipment, even experiments that used similar equipment, still reported varying results. Hence, while it has been demonstrated by previous studies that infants are exposed to ventilator noise in the NICU, we are still unsure of the exact extent of this exposure. In light of this, we aimed to determine whether these variations in ventilator noise could be due to methodological differences by conducting trials measuring noise levels with and without an infant mannequin. Additionally, we examined the effect of positioning and distance of the nasal prongs, as well as the flow rate, on noise levels. The goal was to provide future researchers valuable insight into measuring noise in windy environments, such as ventilators.

This article was previously presented as an abstract at the 2021 American Academy of Pediatrics (AAP) Conference on October 8, 2021.

## Materials and methods

Literature search

The following keywords were used jointly or individually: “ventilator,” “infant,” “hearing,” “development,” “NICU,” “CPAP,” “noise,” “noise level,” and “incubator.” Twenty articles were found and reviewed before conducting this study. However, most reports examined the noise levels in NICUs with a specific focus on ventilation. Nine studies that reported noise levels, the majority of which were above the recommended levels, are summarized in Table 1.

**Table 1:** Previous studies that recorded ventilator noise levels in NICUs NICU: neonatal intensive care unit; HFNC: high-flow nasal cannula; CPAP: continuous positive airway pressure; nCPAP: nasal continuous positive airway pressure; BCPAP: bubble continuous positive airway pressure

Author(s) and Year	Aim	Findings	Method	Noise Levels
Goldstein et al. (2019) [6]	Assessed sound levels of four high-frequency neonatal ventilators	The Dräger VN500 produces less noise than the Sensormedics and Bunnell ventilators	The microphone was positioned where an infant’s head would lie in a neonatal warmer; adjacent to the microphone was a bellows test lung attached to the test ventilator	49.8 dB, 53.6 dB, 54.1 dB, 53.7 dB
Kazemizadeh et al. (2015) [7]	Demonstrated that ventilators can expose patients to noise through bone conduction (BC) and air conduction (AC)	There was concerning ventilator-dependent noise present in ventilation that could be presented via BC	The noise meter was placed in an incubator, and the ventilators were placed 4 ft from where the patient’s head would lie	BC sound levels (74.1, 81.1, 86, 89.2 dBC). AC sound levels (72.8, 72.9, 70, 71.7 dBC)
Roberts et al. (2014) [8]	Studied whether HFNC produced more noise than bubble CPAP	HFNC did not generate more noise than BCPAP	Noise levels were measured in the external auditory meatus using a microphone probe tube	HFNC 49.1 dB was mean 3.0 dBA quiet­er than BCPA 50.7 dBA
König et al. (2013) [9]	Examined noise levels of two HFNC devices compared to a continuous flow CPAP device	Both HFNC devices produced higher noise levels compared to the CPAP device	The microphone of the sound meter was placed 2 cm into a mannequin’s oral cavity in an incubator that was not in operational mode	81.2-91.4 dBA, 78.8-81.2 dBA, 73.9-77.4 dBA
Trevisanuto et al. (2011) [10]	Compared the noise produced by a neonatal helmet CPAP and a conventional nCPAP system, as well as the effect of the gas flow rate	Noise generated by the neonatal helmet CPAP was higher than conventional nCPAP systems. In the helmet, noise depends on the gas flow rate	Helmet CPAP was placed in an incubator, while the prongs were placed in the nose of a mannequin. The exact position of the noise meter was not described	70.0 dB, 62.7 dB
Kirchner et al. (2011) [11]	Determined which CPAP generator creates the least noise	Jet CPAP generators produce more noise than conventional CPAP	The CPAP device was placed in the middle of a closed incubator. The microphone was at a 90° angle 2 mm lateral from the prongs	62 dBA, 55 dBA, 83 dBA, 72 dBA
Cavaliere et al. (2008) [12]	Measured noise intensity during CPAP performed with two interfaces (fa­­­­­ce mask, helmet) and four delivery systems	Maximum noise levels may cause patient discomfort	The microphone was fixed on the right tragus in correspondence with the gas inlet to the helmet	57 dBA, 93/94 dBA
Karam et al. (2007) [13]	Measured the noise levels of various CPAP drivers	nCPAP drivers generate a large amount of noise, often higher than recommended limits	Noise measured in the oral cavity of infants using a microphonic probe with a flexible capillary tube	88.6 (SD: 18.8) dB
Surenthiran et al. (2003) [14]	Determined noise intensities within the ear and post-nasal space on different modes of ventilatory support	High noise intensities in the post-nasal space of individuals receiving CPAP	A portable probe microphone was used for the measurements in infants receiving no respiratory support, CPAP, and conventional ventilation	41.7 dB SPL (NS), 39.5 dB SPL (CV), and 55.1 dB SPL

Study design

All experiments were performed in a research lab outside the NICU to control environmental factors, such as communication. The design of our study was chosen with a view to recreate the settings of a previous experiment in the literature [10]. A ventilator (LEONI PLUS, Heinen + Löwenstein, Bad Ems, Germany) was set up according to the instructions provided in the manual. The ventilator settings were as follows: for the high-frequency (HF) mode, mean airway pressure = 15.0 cmH_2_O, HF Freq = 12 Hz, amplitude = 20.0 cmH_2_O; for nasal continuous positive airway pressure (nCPAP), gas flow = 12 L/min. The mannequin was a life-size intubation phantom of a newborn baby [Coburger Lehrmittelanstalt (CLA 8/58), Coburg, Germany] for orotracheal and nasotracheal intubation with realistic nasal, oral, and pharyngeal cavities.

Noise levels were measured using a Casella CEL 24X noise meter. The device was calibrated using the calibrator (Casella CEL-120/2 for Class 2 instruments) according to the manufacturer's instruction at 114/96 dB at 1000 Hz. The device was set to measure the sound pressure levels (L_eq_) in a range of 30-100 dB using the dBA mode. All data were recorded in five-second increments over two minutes, resulting in 24 data points per measurement. To eliminate artifacts and outliers, such as handling the equipment when starting or stopping the measurement or alarms of the timer at the beginning and the end of the measurement, the data was transferred to a Microsoft Excel sheet where the first and last readings of each trial were excluded. The average, L_Aeq5s_, was determined by first converting the A-weighted decibels for each Δt (5 s) measurement period into absolute intensity values. We then removed potential outliers, such as the first and last measurements of each trial to account for noise not produced by a ventilator. The 5-s average was then calculated and converted back to dBA [15].

**Figure 1:**
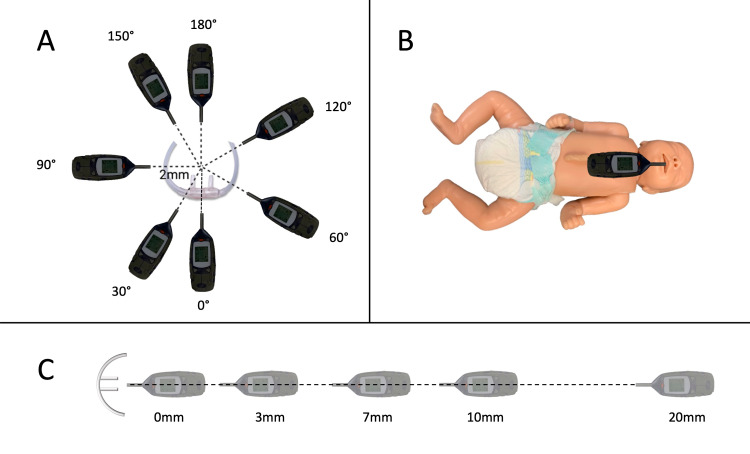
Experimental setup while measuring noise levels (dB) at different angles (A), with a mannequin (B), and at different distances from the nasal prongs (C) dB: decibels

The first trials conducted included measuring noise at different angles between the noise meter and the tip of the prongs, precisely at 0°, 30°, 60°, 90°, 120°, 150°, and 180° (Figure 1A). Two baseline measurements were taken: one with background noise from a refrigerator in the lab, which could not be turned off, and another without noise from the refrigerator. The prongs were threaded through a small hole in the side of the box and were taped to a small box. The small box in which the prongs rested was then taped to the cloth at the bottom of the larger clear box, allowing for airflow to be unhindered during measurements. The ventilator was set to 12 L/min in the nCPAP mode, which was a setting used in the study we aimed to recreate, while the membrane of the noise meter was placed approximately 2 mm laterally from the center of the prongs. For measurements taken at 0° and 30°, a small box was placed underneath the noise meter to allow the membrane to be slightly above the end of the prongs since these angles did not allow for the noise meter to be lateral to the prongs. For all other measurements, the noise meter was placed on the surface of the cloth in the large box to ensure that the membrane was level with the end of the prongs. At 180°, the membrane of the noise meter was facing the end of the nasal prongs. Measurements were also taken using the HF mode with the settings described above.

Trials including measurements in the oral cavity near the soft palate and at the entrance of the mouth of an infant mannequin were also conducted at 12 L/min of gas flow in the nCPAP mode (Figure 1B). A baseline measurement was taken near the chest of the mannequin, pointing towards its head. One two-minute recording taken in the oral cavity was run for four minutes to help eliminate noise from starting and stopping the noise meter, and therefore it was split into two separate measurements during data analysis. Another measurement, run for two minutes, was taken at 30° from the vertical in the oral cavity. Each measurement was recorded in the tables in two-minute intervals for consistency.

In addition, trials were also conducted in the HF mode using varying distances from the nasal prongs. Two new baseline measurements were taken: one with the noise from the refrigerator and one measurement without this background noise. Measurements were taken at 0 mm, ~3 mm, ~7 mm, ~10 mm, and ~20 mm away from the prongs (Figure 1C).

Logarithmic mean values taken for each trial included all data points except the first and last ones to eliminate noise error when data recording was started and stopped. Then, another logarithmic mean value was calculated, excluding all additional outliers, which were likely due to human background noise in the lab, that were noticeably different from the rest of the data points. However, not all trials contained noticeable outliers, and hence a second calculation was not completed in these cases.

## Results

The mean logarithmic noise levels for each of the trials conducted are reported in Tables 2-4. A positive correlation can be demonstrated when examining the mean logarithmic noise levels (dB) at 12 L/min of gas flow in the nCPAP mode without a mannequin in Table 2. At 0°, the mean logarithmic value was 47.6 dB and had risen to 58.2 dB at 180°. The oral cavity and the entrance of the mouth of the mannequin had similar values (Table 3) but were higher than baseline, where the noise was measured with the gas flow turned off. The logarithmic mean baseline measurement was 44.4 dB.

**Table 2:** Mean noise levels (dB) at 12 L/min of gas flow in an incubator without a mannequin, with nasal prongs facing from 0° (same direction directly above prongs) to 180° (directly opposite prongs) dB: decibels; CPAP: continuous positive airway pressure

	0°	30°	60°	90°	120°	150°	180°
CPAP	47.6	46.8	45.7	45.9	47.7	54.2	58.2
High frequency	48.1	47.6	48.5	52.6	52.8	57.0	58.4

**Table 3:** Mean noise levels (dB) at 12 L/min of gas flow with a mannequin, where the noise meter was placed in the oral cavity, at 30° to the vertical in the oral cavity, and in the entrance of the mouth The measurement in the oral cavity was taken over four minutes and divided into two separate two-minute trials (Oral Cavity Trial 1, Oral Cavity Trial 2) dB: decibels; CPAP: continuous positive airway pressure

	Oral Cavity Trial 1	Oral Cavity Trial 2	Oral Cavity at 30° to the Vertical	Entrance of Mouth
CPAP	54.7	51.6	52.7	50.1

**Table 4:** Mean noise levels (dB) at high frequency with the nasal prongs at varying distances from the noise meter without a mannequin dB: decibels

	0 mm	~3 mm	~7 mm	~10 mm	~20 mm
High frequency	72.3	82.2	80.3	77.9	47.6

**Figure 2:**
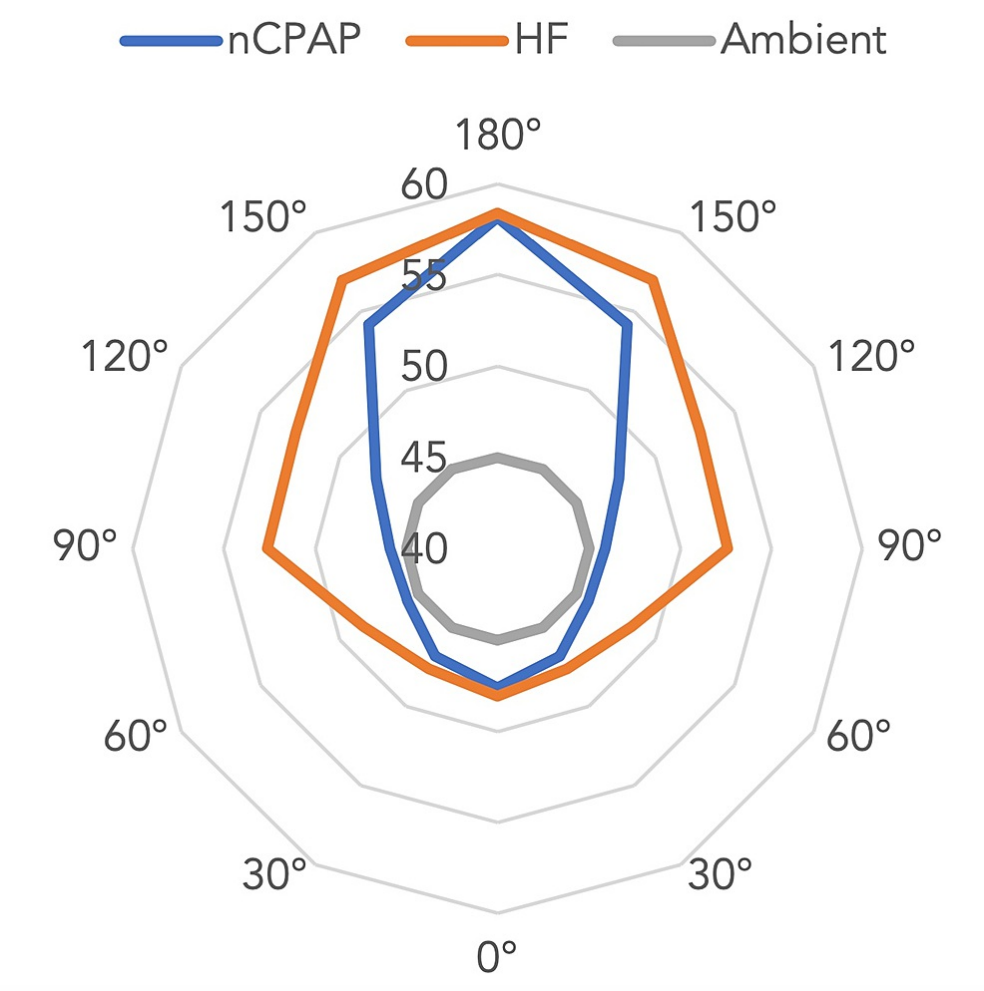
Spider chart representing the noise levels (dB) measured at various angles, where 180° is directly facing the opening of the prongs dB: decibels; nCPAP: nasal continuous positive airway pressure; HF: high frequency

In the HF mode, a positive correlation can also be observed when examining the trend from 0° to 180° (Figure 2). At 0°, the noise level was 48.1 dB, while at 180°, the recorded noise level was 58.4 dB (Table 2). The noise levels measured in the HF mode were also significantly higher than in the CPAP mode. In trials conducted with the mannequin using the nCPAP mode (Table 3), there was a slight difference between the noise level recorded in the oral cavity, 54.7 dB and 51.6 dB, compared to the entrance of the mouth, 50.1 dB (without outliers).

There was no strong correlation between noise level (dB) and distance from the nasal prongs (Table 4), as there was no noticeable pattern. Furthermore, most data points for the measurement taken at approximately 10 mm from the prongs were not recorded by the noise meter as they were too high, which was indicated on the noise meter. However, at approximately 20 mm away from the end of the nasal prongs, the noise level recorded was 47.6 dB, which was much lower than other measurements, with the exception of the baseline.

## Discussion

While it has been previously documented that ventilators produce noise in NICUs, we investigated whether the reported variations could be a result of methodological differences. Settings similar to a previous study were used in order to attempt to recreate and reproduce those results [10]. We explored the effect of distance, nCPAP vs. HF, and nasal prong positioning on noise levels. We conducted trials in both nCPAP and HF modes to compare noise levels between the two to demonstrate that different settings can produce significantly different results. This can be compared to the noise levels measured at different angles. One goal was to demonstrate that noise measured at different distances would be significantly different from each other, and this would likely also differ in the nCPAP mode. There was no discernible pattern, which may be attributed to the production of ambient noise and microphone self-generated noise at different distances. Our results suggest that noise measurements are sensitive to angles and distance, with noise levels ranging from 45.7 dB to 82.2 dB. The differences in our results compared to previous studies on which we based our methods suggest that the methodology of these studies may not be completely reproducible, and wind-induced noise from the ventilator may also impact results.

At first glance, the noise differences found in our study look small but the dBA scale is logarithmic, increasing the dBA value by 10, i.e. from 45 to 55, and increases the sound intensity by 10-fold. The significant changes in noise levels of multiple magnitudes found in our study cannot be explained by varying the positioning and distance slightly but requires further explanation. In acoustical engineering literature, the generation of wind noise caused by air flowing around an object is a well-described phenomenon [16]. It is the turbulence in the airflow that causes pressure changes, which are experienced as noise. Measured wind noise can have two different origins: (1) ambient noise from wind interaction with the environment, such as the nasal interface, or (2) wind-induced microphone self-noise, which is caused by the presence of the device itself. This self-generated noise is an artifact of the noise measurement and depends on the device’s placement and geometry with regard to the airflow [17]. For example, wind-induced self-noise can be experienced when using a phone in a windy environment. By measuring noise in the presence of airflow, i.e. ventilator, it is almost impossible to distinguish between the origin of the two sources. Tests in “silent” wind tunnels have shown that these noise artifacts can easily reach 100 dB even at low wind speed levels (5 m/s) [17]. In the field of environmental noise evaluation of wind turbines, the South Australian Wind Farms Environmental Noise Guidelines 2010 propose that measures be taken to reduce the influence of wind noise on the microphones and to ensure that the reported background noise level is the result of wind-induced ambient noise, rather than wind-induced microphone noise [18,19]. Demonstrating the dependency of distance and angle of the sound meter to the nasal prong while keeping other factors like airflow constant, we showed that our measurement was severely affected by wind-induced artifacts. Slight changes in the settings influenced the results of our measurements and therefore questioned the actual noise level of the ventilator. Our results may have been affected by both ambient noise and microphone self-noise as demonstrated by the differences in measured noise due to slightly varying settings.

Previous literature has reported wide ranges of noise levels, ranging from below the recommended maximum of 45 dB to significantly higher, such as 80 dB or above [6-14]. Furthermore, our results indicate similar trends, with most measurements above 45 dB. Unfortunately, most previous articles do not describe their methodology in extensive detail and neither do they elaborate on preventative measures to reduce wind-generated microphone noise. Therefore, we can only speculate that some results might be influenced by the airflow around the microphone reporting higher noise levels than infants are actually exposed to. The results of this study demonstrate the importance of providing a detailed methodology as well as implementing measures to reduce wind-generated noise.

In a similar study, Lucchini et al. investigated the noise levels of helmet CPAP in adults using different gas inlet systems and gas flow settings [20]. The measured noise levels were compared with the perceived noise levels on a scale of 1-10. The data showed inconsistent results where the device measured loudest was perceived as the least noisy one and vice versa. Since there were no reasons mentioned to explain this paradox, there is thus good reason to suspect that the results were affected by the positioning of the device and the airflow creating a self-generated noise.

There were a number of limitations that we encountered during this experiment. The average ambient noise was measured to be 44.4 dB. The lab in which this experiment was conducted contained a refrigerator that produced noise that we were unable to turn off manually, but would randomly turn off automatically. Hence, a number of baseline measurements were recorded while the refrigerator was off and on before each set of measurements. Therefore, this setting likely contributed to the noise measured in our trials. However, while this setup was not ideal due to environmental noise, this setting is more comparable to a typical NICU as a result. While the baseline noise level recorded was likely not lower than that of a NICU, the variability between measurements would be lower than in a NICU as there would be less natural variation that influenced our measurements. Additionally, we used a mannequin and an acrylic box to simulate an infant and an incubator, respectively, as we were unable to conduct these trials using human participants, potentially leading to less realistic measurements. We also could not report on noise experienced through bone conduction, which would typically occur in infants, as our experiment did not test for this. This study did not measure noise within the ear canal of the newborn mannequin because the noise meter was too large to be placed within the canal. In addition, noise measurements had previously been measured in the ear canal in other published literature [14]. Furthermore, while it may be difficult to standardize noise measurements due to different settings and the availability of equipment, the purpose of our study was to encourage researchers to take into account the different locations and devices when comparing noise levels. For example, it would not be ideal to compare measurements taken in the oral cavity with measurements taken within the incubator without a mannequin without accounting for wind-induced noise. Lastly, the best, or most accurate method for determining how much noise an infant is actually exposed to when on a ventilator cannot be determined by the results of this study. That would likely require a long-term follow-up with newborns to compare measured noise levels to future hearing and sensory development issues, with the quantity and quality of adverse events then being compared against the initial settings.

## Conclusions

There is reason to believe that some findings in recent literature related to noise exposure to ventilated preterm infants might be potentially influenced more by the study design than the ventilator type or setting. Our study found that changes in ventilator settings (i.e. nCPAP vs. HF), and distance and positioning of the nasal prongs could result in significant differences in noise level. These results can likely be attributed to the fact that airflow around the microphone is different depending on the slight changes in the experimental setting. However, we recommend further research and detailed reporting in the NICU in order to better understand the results and their application to future clinical practices. We urge caution with respect to measuring noise in an airflow environment without assessing the risk of self-generated noise using sound-measuring devices. The results of this study can provide future researchers who explore noise measurements in the NICU insights into measuring noise in windy environments, such as ventilators. Future research that measures noise levels must consider the effect of wind-induced noise before providing clinical recommendations, such as specific ventilator settings, to be implemented in the NICU.
